# Nanoscale Control of Structure and Composition for Nanocrystalline Fe Thin Films Grown by Oblique Angle RF Sputtering

**DOI:** 10.3390/ma15176134

**Published:** 2022-09-04

**Authors:** Cristina C. Gheorghiu, Aurelia Ionescu, Maria-Iulia Zai, Decebal Iancu, Ion Burducea, Gihan Velisa, Bogdan S. Vasile, Adelina C. Ianculescu, Mariana Bobeica, Daniel Popa, Victor Leca

**Affiliations:** 1 Extreme Light Infrastructure-Nuclear Physics (ELI-NP), “Horia Hulubei” National Institute for R&D in Physics and Nuclear Engineering, 30 Reactorului Street, 077125 Măgurele, Romania; 2 Faculty of Physics, University of Bucharest, 077125 Măgurele, Romania; 3 Department of Applied Nuclear Physics, “Horia Hulubei” National Institute for R&D in Physics and Nuclear Engineering, 30 Reactorului Street, 077125 Măgurele, Romania; 4 Department of Science and Engineering of Oxide Materials and Nanomaterials, Faculty of Applied Chemistry and Materials Science, University Politehnica of Bucharest, 060042 Bucharest, Romania

**Keywords:** oblique angle sputtering, nanocrystalline Fe films, deposition parameters effect

## Abstract

The use of Fe films as multi-element targets in space radiation experiments with high-intensity ultrashort laser pulses requires a surface structure that can enhance the laser energy absorption on target, as well as a low concentration and uniform distribution of light element contaminants within the films. In this paper, (110) preferred orientation nanocrystalline Fe thin films with controlled morphology and composition were grown on (100)-oriented Si substrates by oblique angle RF magnetron sputtering, at room temperature. The evolution of films key-parameters, crucial for space-like radiation experiments with organic material, such as nanostructure, morphology, topography, and elemental composition with varying RF source power, deposition pressure, and target to substrate distance is thoroughly discussed. A selection of complementary techniques was used in order to better understand this interdependence, namely X-ray Diffraction, Atomic Force Microscopy, Scanning and Transmission Electron Microscopy, Energy Dispersive X-ray Spectroscopy and Non-Rutherford Backscattering Spectroscopy. The films featured a nanocrystalline, tilted nanocolumn structure, with crystallite size in the (110)-growth direction in the 15–25 nm range, average island size in the 20–50 nm range, and the degree of polycrystallinity determined mainly by the shortest target-to-substrate distance (10 cm) and highest deposition pressure (10^−2^ mbar Ar). Oxygen concentration (as impurity) into the bulk of the films as low as 1 at. %, with uniform depth distribution, was achieved for the lowest deposition pressures of (1–3) × 10^−3^ mbar Ar, combined with highest used values for the RF source power of 125–150 W. The results show that the growth process of the Fe thin film is strongly dependent mainly on the deposition pressure, with the film morphology influenced by nucleation and growth kinetics. Due to better control of film topography and uniform distribution of oxygen, such films can be successfully used as free-standing targets for high repetition rate experiments with high power lasers to produce Fe ion beams with a broad energy spectrum.

## 1. Introduction

Throughout the last decades, the intensive use of thin films in particle acceleration experiments using high-power laser systems has led to significant advances in not just applied physics research [1,2], but also in medical science [3,4,5] and industrial applications as well [6,7,8]. Among these, the radiobiological and materials studies performed in ground-based laboratories to discern the effects of cosmic radiation damage suffered by exposed humans and spacecraft components have proven to be quite relevant for future space exploration programs [9,10,11]. The space radiation environment, in which galactic cosmic rays and solar particle events represent common occurrences, consists of heterogenous radiation fields harboring protons, electrons and ions with broadband-energy spectra [12,13,14]. This latter feature has been particularly difficult to mimic using classical accelerator systems, such as linacs and cyclotrons. A more feasible approach is made possible through the use of high-power laser facilities, whose unique beam parameters allow the generation of complex, tunable and multi-energetic radiation environments, akin to the ones encountered in space [10,11,15]. For this purpose, thin film targets might be used as multielement materials to provide the different space-like ionizing particle species mentioned above. The key parameters of these targets can be tuned by optimizing their fabrication procedure and can thus influence the energy distribution of the particle beams produced as a result of their interaction with laser pulses.

A good representative component of galactic cosmic ray’ nuclei with high atomic numbers and energy is iron. Among these heavy ions, iron is potentially the most harmful to the central nervous system [16] on account of its considerable fluence and high atomic number [17]. Iron ion beams have already been generated from the interaction of laser beams with targets consisting of homogeneous pure Fe foils [18] or Al foils containing a layer of Fe contaminants [19]. However, with regard to space radiation experiments, the ion energy must be increased significantly, from the keV range up to the MeV range. One must also take into account that the acceleration of high-Z ions is less efficient than that of lower-Z ion species, and, therefore, in-depth knowledge of the target composition (including the potential for light element contamination) becomes essential for producing high-quality laser-accelerated ion beams that feature controllable energy spectrums [18,20].

Recent numerical and experimental studies have shown that creating nano- and microstructures, as well as patterns on the front surface of the targets, leads to an enhanced laser energy absorption and, hence, an improved conversion efficiency from laser energy into the resulting ion beam energy [21]. A number of surface structures have been proposed to this end, such as gratings [22,23], nanowires [24], nanobrushes [25], carbon foam [26], nanospheres [27,28] or nanoparticles [29]. When used as targets in high-power laser experiments, increasing the effective surface area of thin films through the use of specific growth methods can be an effective way of improving their laser energy absorption capabilities. One way this can be achieved is via the so-called oblique angle deposition (OAD) technique, where the incidence flux makes an angle with the substrate surface normal. The limited surface diffusion, combined with atomic-scale ballistic shadowing give rise to the columnar microstructure of OAD-type films [30,31]. Owing to their internal columnar nanostructure, these particular films are characterized by much larger surface areas compared to those of films grown via the normal configuration, this surface area enhancement being dependent on the deposition angle [30,31,32]. The nanocolumnar structure characteristic of OAD films thus has a direct impact on their physical properties, enabling their use for various scientific applications. To give but one example, anisotropic magnetic properties have been obtained in OAD ferromagnetic films, thereby allowing their application in data storage technology [31].

Many of the multi-element solid targets used to boost the ion acceleration by high-intensity (>10^18^ W/cm^2^) ultrashort (ps-fs) laser pulses use films with surface modulation of the order of the laser wavelength (typically ~800 nm) [21]. There is little understanding of how the acceleration of ionic species in such experiments are affected by the modification of the target’s surface at nanoscale level. Dalui et al. [29] showed that a polished Cu-substrate coated with a thin polycrystalline Cu layer, consisting of average crystallite size of 25–30 nm, can drastically enhance ion acceleration, with the adverse effect on the ion acceleration for crystallite sizes smaller than 20 nm. Another aspect in the multi-elements targets is the screening effect that light elements and their distribution within the target have on the acceleration of heavy-ions. Concentration of the light elements on the surface of the target or their inhomogeneous distribution within the target drastically reduces the energy of the accelerated heavy ions. Therefore, when Fe films are used as multi-element solid targets with nanostructured surface in high-power laser experiments knowledge of the surface topography, grain size and distribution of light elements within the film is critical for efficient ion acceleration. A nanostructured Fe target will have a larger surface with which the laser can interact and thus it is expected that the energy of the Fe ions will be increased [26,29]. In sputtered Fe films, knowledge of the evolution of their nanostructure, morphology, topography and impurity content (mainly oxygen) at different sputter source powers (P_s_), deposition pressures (P_d_), incoming flux directions determined by substrate-to-target distance (d_ts_), and deposition temperatures (T_d_) is still insufficient [33]. There is also a lack of information in the literature on the oxygen distribution within Fe films, a critical aspect for our application. Therefore, the present contribution is aimed to analyze the effect of these sputtering deposition parameters on the structural and compositional properties of Fe thin films grown at room temperature using an oblique sputter configuration. This will allow fabrication of Fe films as potential laser-targets with controllable nanostructure surface and light element distribution through the manipulation of the fabrication process parameters. The results of the study will be applied for fabrication of free-standing Fe targets for multi-species ion acceleration experiments with high-intensity ultrashort laser pulses with the purpose to obtain Fe ion beams characterized by a broad energy spectrum, specific to deep space radiation.

## 2. Materials and Methods

**Figure 1:**
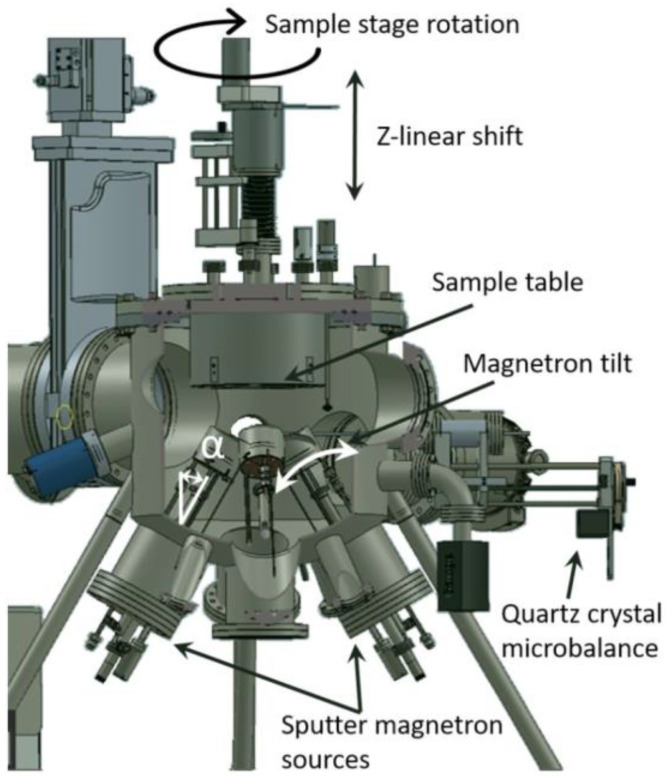
Schematic representation of the deposition chamber used for the sputter deposition of the Fe films, with a cross-section giving an inside view of the deposition chamber, as well as the definition of the tilt angle, α (system drawing by Mantis Deposition Ltd., based on an ELI-NP customized configuration).

The deposition of Fe thin films with thicknesses of 107 ± 22 nm was performed at room temperature (RT) on 10 × 10 × 0.35 mm^3^ (001)-oriented Si substrates (Ted Pella) using a customized ultra-high vacuum cluster system (Mantis Deposition Ltd., Oxfordshire, UK) by means of RF balanced magnetron sputtering [34]. The RF mode of the sputter source, characterized by a stronger magnet designed for the deposition of magnetic materials, was selected as it has the advantage of allowing depositions at lower working gas pressures. By system design, the sputter source makes a tilt angle α (see Figure 1) defined as the angle formed by the plasma direction with the substrate normal. The substrate holder is located above the sputter source, with the sample facing down and the sputter target facing up, as can be seen in Figure 1. The cylindrical magnetrons allow for an in-situ tilt α = 0–65°, operated from outside the vacuum chamber, and feature an integral gas hood and shutter, along with water-cooling. For deposition at different d_ts_ values, the sputter source was tilted at an angle α ~52° for the lowest used d_ts_ value (10 cm), then to α ~38° for the highest used d_ts_ value (16 cm), indicating an oblique sputter deposition configuration. Most of the films were grown at a distance d_ts_ = 13 cm, corresponding to a tilt angle α = 45°. Film thicknesses were measured using an integrated quartz crystal microbalance, calibrated using X-ray reflectometry. An “inside” view of the deposition chamber is given in Figure 1.

Extensive care was taken to ensure a low background pressure before each deposition. To this end, a bake-out from inside the deposition chamber was performed using the sample stage heater, as well as from the outside using electric heating tapes, in order to reach background pressures better than 2 × 10^−9^ mbar. The deposition chamber features a double wall that can be used for cooling the chamber with water, gas or liquid nitrogen, so as to further improve upon the background pressure. A load-lock was used to transfer the samples in order to reduce contamination of the deposition chamber, with the sample transfer into the deposition chamber being performed only after the background pressure inside the load-lock chamber reached better than 5 × 10^−8^ mbar.

Prior to deposition, the substrates were ultrasonically cleaned in acetone and isopropanol (for 10 min each) in order to remove any organic contaminants from the surface, then dried out under a stream of air flow. The Si wafers were used without making any attempts to remove the native SiO_2_ surface layer. Further on, the substrates were securely fixed to a Mo-based holder and introduced in the deposition chamber, where the samples can be placed at different distances to the target. Furthermore, all substrates were pretreated in vacuum (~10^−7^ mbar) for 60–90 min at ~200 °C in order to further reduce the surface contaminants and residues, as well as to recrystallize the native SiO_2_. Next, during the substrate cooling phase down to RT, a target cleaning step (pre-sputtering) was performed for ~15 min, under a pressure of 5 × 10^−3^ mbar Ar. After this cleaning step, the gas was turned off and the film deposition was then performed only after the background pressure recovered to a level of better than 7.5 × 10^−9^ mbar. For sputtering, a commercial Fe target from Kurt Lesker (Hastings, UK) was used, featuring 99.95% purity and dimensions of 3 inches in diameter and 2 mm in thickness. The sputtering process was carried out under an inert gas atmosphere (Ar, 99.999% purity), at deposition pressures (P_d_) in range of P_d_ = (1–10) × 10^−3^ mbar Ar, at RF source powers (P_s_) between 50 W and 150 W, and at several target-to-substrate distances (d_ts_) between 10 cm and 16 cm, respectively. Depositions were performed at RT in order to avoid the formation of iron silicides that occurs at higher temperatures [35]. During deposition, the temperature measured by a thermocouple placed behind the sample holder reached about 35 °C. However, the actual temperature of the substrate/film surface is expected to be higher. The deposition parameters used for the Fe films fabricated in this work are summarized in Table 1.

The nanostructure of the films was characterized by X-Ray Diffraction (XRD) and, for a few selected films, also via High-Resolution Transmission Electron Microscopy (HR-TEM). Surface topography and morphology were investigated using Atomic Force Microscopy (AFM) and Scanning Electron Microscopy (SEM), whereas the elemental distribution in the films was determined through the Energy Dispersive X-ray Spectroscopy (EDS) and Non-Rutherford Backscattering Spectroscopy (NRBS) techniques.

The XRD measurements were performed using an automated multipurpose high-resolution Rigaku SmartLab X-ray diffractometer (Rigaku Corporation, Tokyo, Japan) featuring a 9-kW rotating anode (Cu-Kα1 radiation, λ = 1.5406 Å), a 5-axis goniometer and a high-resolution HyPix-3000 hybrid pixel array detector. The SmartLab Studio software was employed for data analysis. The grazing incidence 2θ scans (with the incident X-ray beam angle, ω, set at 1°) were acquired without the use of monochromators, with the X-ray tube being operated at 45 kV and 100 mA. The films were scanned in steps of 0.005° using a scanning speed of 0.5°/step, in a 2θ range of 40–85°. In order to obtain a highly convergent X-ray beam, several slits were placed along the beam axis. The incidence (primary) beam optics consisted of a cross beam optics (CBO) unit, a 5° incidence parallel soller slit, a motorized 0.05 mm incidence slit (for limiting the height of the X-ray beam and, therefore, its projection on the sample surface at grazing incidence angles), and a manually-placed 2 mm long limiting slit (for controlling the width of the X-ray beam). The receiving (secondary) beam optics consisted of a 20 mm receiving slit, a 0.5° parallel slit analyzer (PSA) and a 5° receiving parallel soller slit. 

The AFM studies were performed using an NT-MDT NTEGRA system in semi-contact mode, in air, using a Si tip coated with W_2_C (34 N/m force constant, 380 kHz resonant frequency, tip curvature radius <10 nm, Au reflective side coating). AFM data analysis was performed using both the integrated analysis software (Nova Px SPM Control Program v.3.4.1., NT-MDT Co., Moscow, Russia) as well as the WSXM software (WSxM v4.0, Nanotec Electronica S.L., Madrid, Spain) [36], but the latter was the one primarily used for analyzing the AFM data presented in this work. The SEM investigations were carried out with an ultra-high resolution Tescan MAIA-3 field-emission electron microscope, operated at 15 kV beam accelerating voltage, with the images being recorded using an In-Beam Secondary Electron detector. A Titan Themis 200 image corrected transmission electron microscope (FEI), equipped with a high-brightness field emission gun (X-FEG) set to 200 kV was used for the HR-TEM studies. A Super-X Energy Dispersive Spectroscopy detector was used for acquiring elemental maps of the samples, while the Selected Area Electron Diffraction (SAED) patterns were acquired in TEM mode. The samples for HR-TEM studies were prepared by a combination of mechanical polishing and ion beam milling. Details of the HR-TEM samples preparation can be found in Ref. [37]. 

The EDS studies were performed using a silicon drift, Peltier cooled Bruker QUANTAX 200 XFlash 6/30 light element detector (Bruker Nano GmbH, Berlin, Germany), with an energy resolution ≤126 eV at MnKα. The integrated Bruker ESPRIT Quant v 2.3 quantification software (Esprit 2.3, Bruker Nano GmbH, Berlin, Germany) was used for performing standardless, semiquantitative EDS spectra analysis. Several films were further analyzed via NRBS in order to determine the film thickness and the distribution of elemental composition within the films. The NRBS experiments were conducted using two ion beam energies (3.043 MeV and 3.075 MeV, respectively), allowing the quantification of oxygen distribution, as a contaminant, on both the surface and within the deposited layer, alongside the Fe concentration. In order to check the presence of other light contaminants on the surface (e.g., H and C), the higher energy of alpha particles was used to take advantage of the resonant conditions for C, at 4.282 MeV. For profiling H, Foil–Elastic Recoil Detection Analysis (Foil-ERDA) was performed using 1.5 MeV alpha particles. The NRBS and Foil-ERDA measurements were conducted using the instrumentation available at the ion beam analysis (IBA) reaction chamber of a 3 MV Tandetron accelerator, developed by High Voltage Engineering Europa B.V. (HVE) [38]. This reaction chamber is equipped with a three-axis (phi, theta, and tilt) computer-controlled goniometer enabling the precise orientation of samples during IBA measurements. In addition, the IBA chamber is also equipped with both fixed (165°) and movable (between 10° and 150°) solid-state charged particle detectors. It is important to mention that in front of the movable NRBS detector there is a six-position foil-changer. The fixed Si detector was used for the NRBS measurements, while for the Foil-ERDA measurements, the energy of the hydrogen recoils was measured by the movable Si detector, positioned in the forward direction at 30° with respect to the beam normal. The incident beam angle and the exit angle, as measured from the normal to the sample surface, were both 75°. A 6 μm thick Mylar absorber foil was located in front of the movable detector so as to stop all scattered He ions. Please note that all NRBS experiments were performed in a random direction (by tilting the samples at an angle of 7° in reference to the surface normal), in order to minimize the channeling effects. The energy resolution of the experimental setup was about 18 keV for the NRBS and 35 keV for the Foil-ERDA measurements. Simulations of the NRBS spectra were performed using the SimNRA software package (SimNRA v 7.02, Max Planck Institute for Plasma Physics, Garching, Germany) [39].

## 3. Results and Discussion

### 3.1. Microstructural Analysis

**Figure 2:**
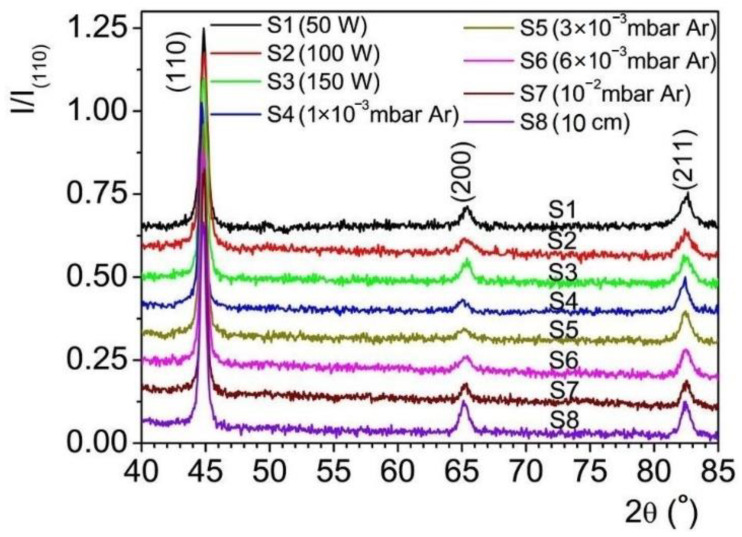
XRD profiles of the Fe thin films grown on (100) Si substrates, indicating the polycrystalline structure of the films. The deposition parameters corresponding to each film, marked S1–S8, can be found in Table 1.

Figure 2 shows the XRD spectra obtained for the Fe thin films deposited on (100) Si substrates under different deposition conditions (P_d_, P_s_, and d_ts_) and the calculated structural parameters of the films are summarized in Table 2. The peak correspondent to the (110) growth direction of each spectrum was fitted analytically using the Voigt function [40,41] in order to determine the crystalline coherent length (crystallite size), D_ef_, using relation (1), as well as the microstrain (eps) or mean-square strain <ε^2^>^1/2^, using relation (2):

(1) $$D_{\mathrm{ef}}=\frac{0.9\lambda }{\beta_{L}\cos\theta }$$

(2) $$\mathrm{eps}=\langle \epsilon^{2}\rangle {}^{1/2}=\frac{\beta_{G}}{2\cdot \sqrt{2\pi }\cdot \tan\theta }$$

where λ is the wavelength of the Cu radiation, β_L_ is the integral breadth parameter of the Lorentz component of the Voigt profile, θ is the (110) peak position and β_G_ is the integral breadth parameter of the Gaussian component of the Voigt profile [42]. The integral breadth parameters, β_L_ and β_G_, were calculated using the Lorentzian and Gaussian FWHM (full width at half maximum) values for each (110) peak resulted from the Voigt profile fitting. The film unit cell parameter, *a*, was calculated via the formula described by the Laue condition (3), using the (110) peak position, θ:

(3) $$h^{2}+k^{2}+l^{2}={\left(\frac{2\cdot a\cdot \sin\theta }{\lambda }\right)}^{2}$$

The ratio A_(hkl)/_A_total_ of the peaks’ area (Table 2) was used as a direct indication of the amount of each growth orientation, with A_(hkl)_ being the peak area of a specific (hkl) direction, and A_total_ = ΣA_(hkl)_, where (hkl) are (110), (200), and (211), respectively.

All films showed a polycrystalline structure, with a (110) preferential orientation (Figure 2) and the degree of polycrystallinity being dependent on the deposition conditions. This type of structure is characteristic to OAD Fe films, grown on Si substrates [43], as well as when a normal configuration is used for deposition [44]. The film unit cell parameter (Table 2) was calculated to *a* ~2.859 ± 0.004 Å, which is close to the bulk Fe cell parameter, *a*_0_ = 2.86 Å (PDF2 Card No. 00-001-1252) [45]. The analysis of the (110) peaks showed that the crystallite size in this crystallographic direction, D_ef_, slightly increased with the RF source power (samples S1–S3), giving an indication of the improved crystalline quality of the films with increasing RF source power (Table 2). Notably, there was also an increase in the crystallite size with the deposition pressure (samples S4–S7), while decreasing the target-to-substrate distance resulted in an increase of the crystallite size as well (samples S5 and S8). The data in Table 2 indicate that, except for sample S8, the increase of the (110) crystallite size can be correlated to a decrease in the amount of the secondary growth directions, (200) and (211), respectively. This reduction in the amount of secondary growth directions is more noticeable at the highest used deposition pressure (10^−2^ mbar Ar). Therefore, the increase of the crystallite size in the (110) direction seems to be inhibited by these secondary growth directions present within the films, with a higher impact being attributed to the (200) growth direction. However, sample S8—characterized by the shortest d_ts_ and, therefore, the highest deposition rate and the highest average angle of incidence of the adatoms—demonstrated the highest concentration of secondary growth directions. Therefore, the S8 film showed the highest degree of polycrystallinity, whereas the S7 film, grown at the highest deposition pressure (10^−2^ mbar Ar), was characterized by the lowest degree of polycrystallinity. As for the RF source power values used throughout this work, they were revealed to have a modest impact on the degree of polycrystallinity. 

The results of the XRD studies indicated that a high deposition rate paired with a high average angle of incidence of the adatoms seemed to promote a higher degree of polycrystallinity, while a high deposition pressure promoted a (110) preferential orientation for the resulting Fe thin films.

The evolution of the microscopic mechanical stresses in the films’ preferential direction, eps, as calculated with relation (2), and of the macroscopic stresses in the same growth direction, ε_rel_, with ε_rel_ = (*a* − *a*_0_)/*a*_0_, where *a* represents the calculated unit cell parameter from the (110) peak and *a*_0_ the bulk Fe unit cell parameter, is shown in Table 2. Both types of stress were found to be relatively small and independent of the deposition parameters. However, while there was only a small variation of the microscopic stress, eps, with the deposition conditions, the macroscopic stress, ε_rel_, showed a larger variation, with the smallest value being registered for sample S8 (corresponding to the shortest d_ts_ used) and the largest macroscopic stress being noted for samples S1 and S7, corresponding to the lowest P_s_ and, respectively, the highest P_d_ values used in the present work.

An increase in P_s_, P_d_ and/or d_ts_ determined a decrease in the average angle of incidence of the adatoms arriving on the substrate (i.e. the trajectory of the adatom flux to the substrate normal) [32]. As shown in Table 2, the formation of the secondary growth directions follows the same trend, with their concentration decreasing with increasing P_s_, P_d_ and d_ts_ values, the deposition pressure parameter exhibiting the largest influence in this regard. Increasing the RF power source and the deposition pressure also results in an increased deposition rate, having a similar effect with decreasing d_ts_, but with a higher concentration of nucleation sites for reduced d_ts_ due to the increased deposition rate. Therefore, a strong increase in the deposition rate by decreasing d_ts_ results in an increase in secondary growth directions, as compared to the smaller increase in the deposition rate resulting from increasing the P_s_ and P_d_ parameters. The above conclusions show that the constant factor determining the concentration of secondary growth directions is the average angle of incidence of the adatoms arriving on the substrate. For the two samples situated at the extreme sides of polycrystallinity (S7 and S8, respectively), the main difference is given by the average angle of incidence of the adatoms and their kinetic energy, with highest average angle of incidence of the adatoms being registered for shortest d_ts_ value and the lowest kinetic energy for the film grown at the highest pressure. Therefore, the formation of the (110) growth direction is influenced and favored by a lower kinetic energy of the adatoms combined with a low average angle of incidence of the adatoms arriving on the substrate. Once the average angle of incidence of the adatoms is increased, the other growth directions can develop more readily, resulting in an increased degree of film polycrystallinity. Finding the right conditions that kinetically favor the (110) growth direction might enable the fabrication of epitaxial Fe films on the (100) Si substrate even at RT, albeit characterized by a random initial nucleation stage. However, the oblique angle sputtering approach seems to favor polycrystallinity [43].

### 3.2. High Resolution Transmission Electron Microscopy

**Figure 3:**
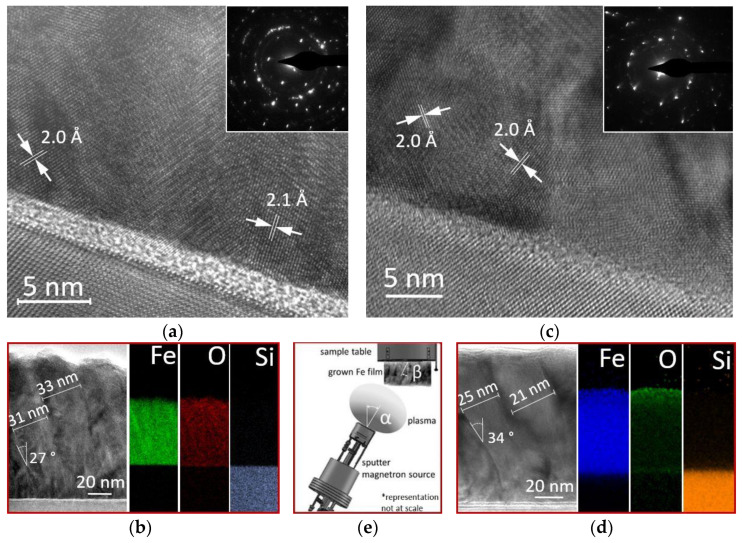
HR-TEM micrographs, inserted SAED patterns (**a** and **c**), and elemental distribution maps of iron, oxygen, and silicon (**b**,**d**), for two Fe films deposited at different RF source powers, P_s_: (**a**,**b**) 50 W (film S1), (**c**,**d**) 150 W (film S3). The deposition parameters were: P_d_ = 6×10^−3^ mbar Ar, d_ts_ = 13 cm, T_d_ = 35 °C. In (**e**), the representation of the nanocolumns tilt angle, β, in relation to the plasma direction angle, α.

The films grown in an off-axis configuration, as is the case in oblique angle sputtering, are usually developed as nanocolumns, tilted away from the substrate surface normal [32,46,47]. This type of nanostructure can then be correlated to the evolution of the film morphology and topography with the deposition parameters. Figure 3 illustrates the results of cross-section HR-TEM analysis for two films deposited at different RF source powers (50 W, in Figure 3a,b, and 150 W, in Figure 3c,d), while the other deposition parameters were fixed (P_d_ = 6 × 10^−3^ mbar Ar, T_d_ = 35 °C, and d_ts_ = 13 cm, corresponding to a plasma tilt angle α ~45°).

The cross-sectional micrographs provided data about the interfaces, grain boundaries and lattice parameters of the analyzed films. Information about the structure of the crystal phase, along with the corresponding crystallographic orientations were obtained from the selected area electron diffraction patterns (SAED, insets in Figure 3a,c) taken from the cross-sections. Moreover, the cross-sectional view provided information on the film thickness and substrate interface quality, while the elemental mapping analysis (Figure 3b,d) showed the elemental distribution in the Fe films.

The HR-TEM micrograph of the sample deposited at P_s_ = 50 W (Figure 3a) showed a structure characteristic of a polycrystalline film, with the crystallites having different orientations (mosaic structure) and demonstrating good overall crystallinity as indicated by the ordered rows of atoms inside the grains. The bright field low-magnification TEM image (Figure 3b, left side) indicated a film with a thickness of about 110 nm, featuring a nanocolumnar grain structure (32 nm average column width), the columns forming a tilt angle β ~27° with the substrate normal. The interplanar distance of 2.0–2.1 Å of most of the grains, corresponding to the (110) planes of the Im-3m bcc (body-centered cubic) structure of Fe (ICDD 00-001-1252), confirmed the XRD data, indicating a (110) preferred growth orientation. A highly disordered interface layer between the film and the silicon substrate was clearly observed (Figure 3a), representing the native SiO_2_ being about 3.5 nm in thickness. The SAED pattern (Figure 3a, inset) confirmed the polycrystalline structure of the film, with the concentric diffraction rings being identified as Fe featuring the same Im-3m bcc structure. The compositional elemental mapping (Figure 3b, right side) showed a homogeneous distribution of Fe throughout the entire sample thickness and a relatively high concentration of oxygen.

The HR-TEM and SAED data for the sample deposited at P_s_ = 150 W are presented in Figure 3c,d, respectively. The approximately 80 nm-thick film showed similar characteristics to the one grown at 50 W, including polycrystallinity, columnar growth and a (110) preferential growth orientation. However, by increasing the RF source power, the 150 W deposited film demonstrated better crystallinity, with well-defined grains and narrower columns (23 nm average column width) which formed higher tilt angles with the surface normal (β ~34°). The HR-TEM data also indicated a smaller tilt angle of the nanocolumns at the beginning of growth (Figure 3d), most probably due to the anisotropic nature of shadowing [30,31,32]. The elemental mapping (Figure 3d, right side) indicated a lower bulk concentration of oxygen (as compared to the 50 W film) with a higher amount of this impurity being concentrated at the surface of the film. The correspondence between the plasma direction angle, α, and column tilt angle, β, is represented in Figure 3e.

As the RF source power was increased, the average energy of the sputtered metal adatoms increased, meaning that the incoming particles were reaching the surface with higher average energies. Furthermore, increasing the RF source power also resulted in more plasma directionality due to less scattering of the incoming particles in the plasma plume [32]. These effects produced films featuring nanocolumns with higher tilt angles (i.e., closer to the plasma direction) with regard to the substrate surface normal, as confirmed by the results presented in Figure 3d. Additionally, an increase in the number of nucleation sites was expected with increased RF source power and, correspondingly, a higher number of smaller islands featuring nanocolumns with smaller diameters, as confirmed by the HR-TEM data (Figure 3b,d).

There are several models that describe the correlation between the plasma direction angle, α, and the nanocolumn tilt angle, β [48,49,50,51]. For the sample grown at 150 W, the ballistic model for columnar growth orientation [51], represented by the correlation relation β = α−arcsin[(1−cos α)/2], gave a nanocolumn tilt angle of ~37°, which is close to the value of 34° observed via HR-TEM. Similarly, for the sample grown at 50 W, the tangent rule [48], represented by the relation tan α = 2 tan β, gave a calculated tilt angle of ~26.5° that agrees well with the experimental value of 27° also resulted from HR-TEM. These results indicate a dependence of the columnar structure on the growth parameters and, possibly, different mechanisms governing the columns’ growth.

Regarding the oxygen distribution in these two films, the results obtained using elemental mapping (Figure 3b,d) do not necessarily represent the actual oxygen distribution across the as-deposited films. This difference may stem from differences in oxygen reactivity between these two samples during their preparation for HR-TEM analysis, the relatively high oxygen concentration in the 50 W sample possibly indicating a higher sensitivity of this film to oxygen intake. Therefore, spectroscopy measurements were performed in order to determine the actual oxygen composition and its distribution in the as-deposited films, the results of which will be presented next.

### 3.3. Surface Morphology and Topography Analysis

**Figure 4:**
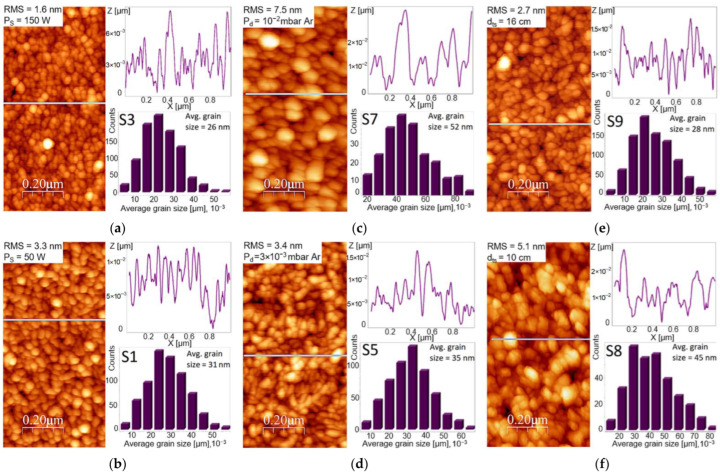
Evolution of surface morphology and topography of Fe films grown on (100) Si with RF source power, P_s_, for (**a**) 50 W and (**b**) 150 W; deposition pressure, P_d_, for (**c**) 3 × 10^−3^ mbar Ar and (**d**) 10^−2^ mbar Ar; target-to-substrate distance, d_ts_, for (**e**) 16 cm and (**f**) 10 cm. The deposition parameters were (**a**,**b**) P_d_ = 6 × 10^−3^ mbar Ar, T_d_ = 35 °C, d_ts_ = 13 cm; (**c**,**d**) P_s_ = 125 W, T_d_ = 35 °C, d_ts_ = 13 cm; (**e**,**f**) P_s_ = 125 W, P_d_ = 3 × 10^−3^ mbar Ar, T_d_ = 35 °C.

Figure 4 shows the AFM images of the Fe films grown on (100) Si substrates, indicating the evolution of the surface morphology and topography with the main deposition parameters (P_s_, P_d_, and d_ts_). All of the films showed a relatively homogeneous surface topography, with their average grain size, root mean square (rms) roughness and grain size distribution being dependent on the deposition conditions. Average grain sizes varied from ~26 nm (Figure 4a) up to ~52 nm (Figure 4c), with the measured rms surface roughness ranging from ~1.6 nm (Figure 4a) to ~7.5 nm (Figure 4c), thus following the trend of the average grain size.

For the RF source power values applied in this study, the Fe films showed relatively flat surfaces (Figure 4a,b), with rms values in the 1.6–3.3 nm range and a smoother surface for higher RF source powers. The surface morphology did not change significantly, but there was an increase in the average grain size with lower RF source power, from ~26 nm at 150 W, up to ~31 nm for 50 W, combined with an important increase in surface roughness, the rms value also increasing from 1.6 nm to 3.3 nm, respectively (Figure 4a,b). The results of the AFM analysis were in concordance to the HR-TEM data, the island dimensions following the nanocolumn width. The impact RF source power had on surface morphology can be explained through an increased kinetic energy of the adatoms and a corresponding higher concentration of nucleation sites, owing to the accelerated deposition rate that comes with higher RF source powers. As a result, the adatoms can migrate easily across the surface and incorporate at the neighboring islands, with the smaller islands concentration increasing proportionally with the deposition rate, resulting in a surface with reduced roughness and a smaller grain size. Therefore, when analyzing the influence exerted by the RF source power on the final surface morphology and topography, there is a combined role of the kinetic energy of the arriving adatoms and the deposition rate which gives rise to nucleation sites.

Varying the deposition pressure demonstrated a stronger impact on the surface topography (Figure 4c,d), the surface roughness drastically increasing at 10^−2^ mbar Ar. The rms values showed an increase in surface roughness from ~3.4 nm for the film deposited at 3 × 10^−3^ mbar Ar, up to ~7.5 nm for the film grown at a pressure of 10^−2^ mbar Ar. Similarly, an increase was observed in the average grain size, from ~28 nm up to ~52 nm, which correlated with the increasing deposition pressure. This results in a decrease in the average energy of the adatoms, thereby reducing the adatom surface diffusion, while simultaneously increasing the scattering of the metal flux in the plasma and decreasing its directionality. Due to their low mobility, the adatoms thus attach to the existing islands at their landing site, resulting in higher surface roughness. This change in the adatoms’ energy and incidence angle triggers a change in the dominant film growth mechanism with pressure, from ambient flux dependent deposition at higher pressures, to directional ballistic shadowing deposition at lower deposition pressures. Both of these growth mechanisms are typically observed for oblique growth configurations [30,32].

Regarding the impact of d_ts_, when the target-to-substrate distance is reduced from 16 cm (Figure 4e) to 10 cm (Figure 4f), there is a small increase in the average grain size from ~35 nm to ~45 nm, with rms also rising in tandem from ~2.7 nm to ~5.1 nm, respectively. By decreasing the d_ts_ value, one gives rise to a cumulative effect of increased kinetic energy, increased deposition rate and, due to the oblique growth configuration, an increase in the scattering angle of the adatoms arriving on the surface, paired with a reduction in their directionality. All of the above effects, when combined with a very low surface mobility due to RT deposition and a limited diffusion time due to the high deposition rate, give rise to increased grain sizes (with larger aspect ratio) and a corresponding increased surface roughness when d_ts_ decreases.

**Figure 5:**
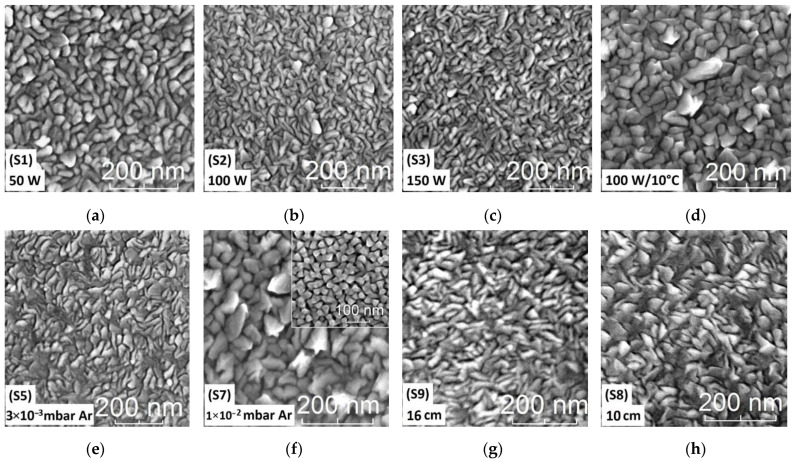

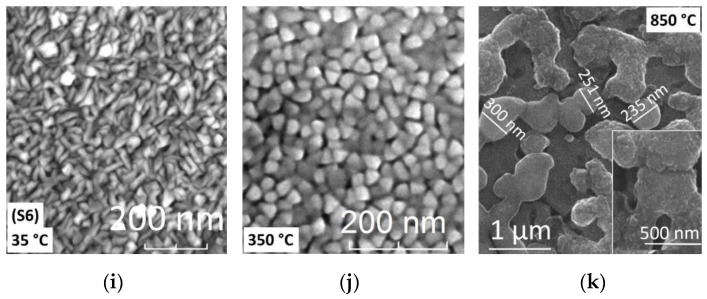
SEM micrographs of the Fe films deposited on (100) Si showing the influence of different deposition parameters on film morphology (**a**–**d**) RF source power (P_s_): (**a**) 50 W, (**b**) 100W, (**c**) 150 W, and (**d**) 100W (with N_2_ cooling). Please note the larger magnification in (**a**). (**e**,**f**) working pressure (P_d_): (**e**) 3 × 10^−3^ mbar and (**f**) 1 × 10^−2^ mbar; inset: on 100 nm Au coated (100) Si; (**g**,**h**) target-to-substrate distance (d_ts_): (**g**) 16 cm and (**h**) 10 cm; (**i,j**) substrate temperature (T_d_): (**i**) 35 °C, (**j**) 350 °C, (**k**) film from (**i**) after annealing for 3 h at 850 °C and 10^−5^ mbar. The deposition parameters were (**a**–**d**): P_d_ = 6 × 10^−3^ mbar, d_ts_ = 13 cm, T_d_ = 35 °C; (**e**,**f**): P_s_ = 125 W, d_ts_ = 13 cm, T_d_ = 35 °C; (**g**,**h**): P_s_ = 125 W, P_d_ = 3 × 10^−3^ mbar, T_d_ = 35°C); (**i**–**k**): P_s_ = 100 W, P_d_ = 6 × 10^−3^ mbar, d_ts_ = 13 cm.

The morphology of the Fe films (i.e., grain size, distribution and shape), as pertains to the mobility of the adatoms on the surface, was determined further by means of SEM analysis (see Figure 5). While the films showed similar morphologies (Figure 5a–c), featuring irregular elongated structures with dimensions of about 30 nm in width and up to 70 nm in length for P_s_ = 150 W, the SEM micrographs indicated a reduction in the grain size with higher RF source power values. This can be explained through the increased kinetic energy of the adatoms, resulting in an increased surface mobility, combined with a proportional increase in the deposition rate with increasing RF source power. A higher deposition rate results in an increased number of nucleation sites, while the increased mobility seen at higher kinetic energies allows the adatoms to find and attach sooner to a neighboring island, the resulting film surface thus developing as an increased number of smaller, flatter islands. These explain the larger grain size observed for the film grown at 50 W (Figure 5a), as compared to the 100 W (Figure 5b) and 150 W films (Figure 5c), respectively.

A reduction in the substrate temperature during deposition (down to 10 °C), achieved through the use of liquid N_2_ for cooling the vacuum chamber, had a similar effect as using a lower RF source power, leading to the formation of large grains, as can be seen in Figure 5d. In this case, the formation of 3D islands was further enhanced by the reduced temperature, resulting in a very low surface mobility of the adatoms. A similar behavior to the one observed with the decreasing temperature was obtained by increasing the deposition pressure from 3 × 10^−3^ mbar Ar (Figure 5e) to 10^−2^ mbar Ar (Figure 5f), which resulted in films with larger grains and lead to a randomly distributed structure with increased roughness, in accordance with the AFM results (Figure 4c,d).

The inset in Figure 5f shows the impact on the film morphology when a 100 nm Au buffer layer is used, with the rest of the deposition parameters remaining the same as for the deposited Fe film shown in the main micrograph of Figure 5f. A smaller grain size was observed in the case of the film grown on Au-buffered (100) Si, with a more uniform distribution, as well. This suggested Fe adatoms exhibit a lower surface mobility on Si than on Au surfaces under the same deposition conditions, most probably due to the presence of the thin native SiO_2_ layer (as shown by the HR-TEM data).

As indicated in Figure 5g,h, the use of larger d_ts_ values during the deposition process resulted in smaller grains being obtained due to the reduced scattering of the adatoms on the deposition surface. A notable, continuous transformation of the surface morphology of the films was observed when increasing the substrate temperature during deposition (Figure 5i–k). The surface evolved from one with elongated grains at 35 °C (Figure 5i), to one showing a more uniform size distribution featuring square-like grains of about 30 nm in width at 350 °C (Figure 5j). A significant change in surface topography was observed after annealing the Fe films in vacuum (10^−5^ mbar) at 850 °C for 3 h (Figure 5k). The aggregation of the Fe particles generated large heterogeneous grains, with some reaching sizes larger than 300 nm as they grouped into larger structures. This latter case also gave rise to the formation of silicates at the Fe-Si interface, as indicated by the XRD data (not shown here).

There are several conclusions that can be drawn from the morphology and topography analyses performed via AFM and SEM. For the deposition parameters used in this study to grow Fe films on (100) Si substrates, the film morphology and topography were determined by the deposition kinetics and the nucleation occurring at the surface. The deposition temperature and gas pressure parameters had the largest impact on the film grain size and distribution, as well as surface roughness. On the other hand, the influence on surface morphology and topography was smaller when varying the RF source power and target-to-substrate distances. The AFM and SEM results indicated a predominantly Volmer-Weber (3D island) growth mode, with the interaction between the adatoms being greater that their interaction with the substrate, growth mode generally observed for metals deposited at low temperatures [31,52,53].

### 3.4. Spectroscopic Studies

**Figure 6:**
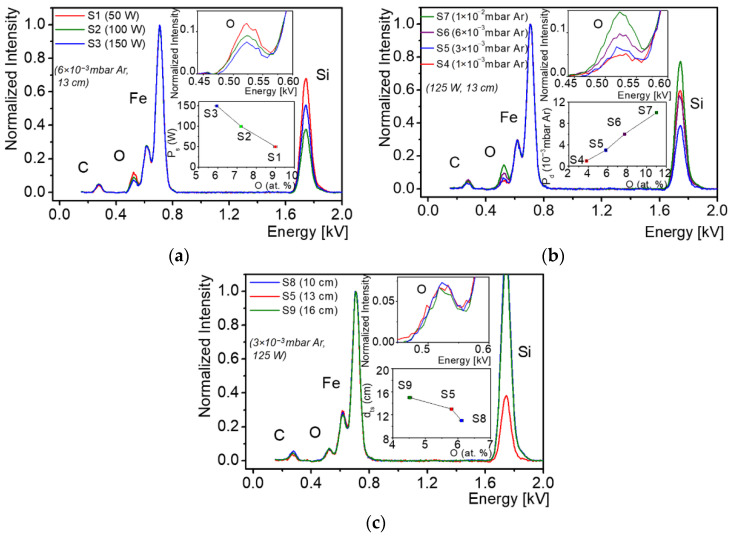
The EDS spectra of the Fe films, showing the evolution of the elemental composition with (**a**) RF source power, P_s_, (**b**) deposition pressure, P_d_, and (**c**) target-to-substrate distance, d_ts_.

In order to determine the evolution of oxygen, as an impurity, with the changing deposition parameters, each film was analyzed by EDS immediately after its growth phase, in order to avoid further surface contamination. The data from the EDS studies, which allowed for a fast, semi-quantitative analysis of the elemental composition of the examined films, was used to better understand the critical deposition parameters that might have a significant impact on the concentration of oxygen impurities. Carbon, which was also detectable by EDS, was instead considered a surface contaminant. The EDS measurements were performed using an accelerating voltage of 7.5 kV (optimized for light elements), a beam intensity of 10 nA and a take-off angle of 30°. The EDS studies results are provided in Figure 6 and Table 3. The composition of each film represents the average of three different data points, with an analysis area of 100 × 100 µm^2^ per point. The EDS peak spectra were fitted with the ESPRIT Quant quantification software, while the net intensity of the X-ray Kα lines of C, O, Si and Fe, plus the Lα line for Fe, were determined after removing the background. 

An overview of the measured EDS spectra for the Fe films, showing the evolution of the film elemental composition as a function of the deposition conditions, is presented in Figure 6. The data showed a steady increase in the oxygen concentration of the films with decreasing RF source power, P_s_ (Figure 6a), and target-to-substrate distance, d_ts_ (Figure 6c), or with increasing deposition pressure, P_d_ (Figure 6b), although the extent of their effects were quite different. The largest impact on the oxygen composition of the films was given by the deposition pressure (directly related to the Ar gas flow), followed by the RF source power and an even lesser impact elicited by the target-to-substrate distance parameter. According to the EDS data presented in Table 3, the lowest oxygen concentration (~4 at. %) was detected in the films grown using the lowest deposition pressure (1 × 10^−3^ mbar Ar), while the largest amount of oxygen (~11 at. %) was measured in the films grown at the highest deposition pressure (1 × 10^−2^ mbar Ar). Higher RF source powers and substrate-to-target distances, combined with a low deposition pressure, can therefore be used to obtain the lowest possible concentration of oxygen impurities in the films. It bears mentioning that the EDS values for oxygen concentration represent an average of the probed volume of the examined film, including its potential surface contamination, as well as the amount stemming from the SiO_2_ interface. 

**Figure 7:**
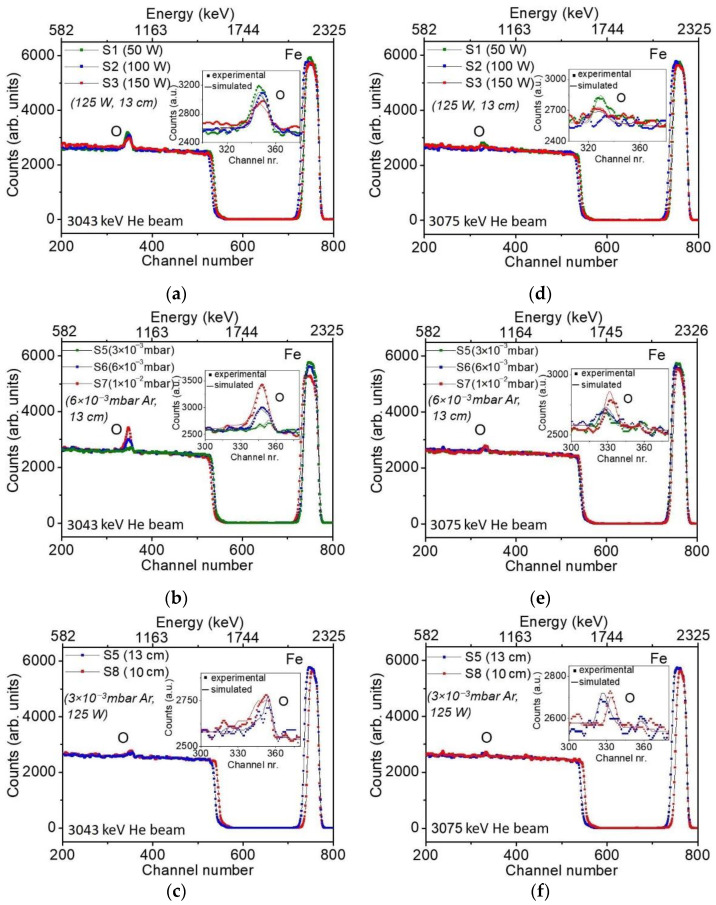
NRBS spectra at 3043 keV (**a**–**c**) and at 3075 keV (**d**–**f**) for the Fe films grown on (100) Si, showing the evolution of elemental composition with (**a**,**d**) RF source power, P_s_, for P_s_ = 50 W, 100 W, and 150 W; (**b**,**e**) deposition pressure, P_d_, for P_d_ = 3 × 10^−3^ mbar Ar, 6 × 10^−3^ mbar Ar and 1 × 10^−2^ mbar Ar; (**c**,**f**) target-to-substrate distance, d_ts_, for d_ts_ = 10 cm and 13 cm, respectively. The film deposition parameters were (**a**,**d**) P_d_ = 6 × 10^−3^ mbar Ar, T_d_ = 35 °C, d_ts_ = 13 cm; (**b**,**e**) P_s_ = 125 W, T_d_ = 35 °C, d_ts_ = 13 cm; (**c**,**f**) P_s_ = 125 W, P_d_ = 3 × 10^−3^ mbar Ar, T_d_ = 35 °C.

The quantitative elemental composition of the films, their depth profiles and the resulting total film thicknesses were determined via NRBS measurements, with the results presented in Table 3 and Figure 7.

In ion beam analyses, the areal density or thin film units (TFU) are used, since the energy loss is measured in eV/(atoms/cm^2^), with one monolayer typically containing in the order of 10^15^ atoms/cm^2^ [54]. Therefore, the depth scale is given in TFU units [55], where 1 TFU = 10^15^ atoms/cm^2^. In order to profile the film thicknesses, two energies were used for measuring the oxygen composition (3.043 MeV and 3.075 MeV). The higher energy was chosen to give the maximum resonance on oxygen and thereby profile the inner half of the film. The statistic of the NRBS measurements was 50 µC.

The experimental NRBS data were simulated using the SIMRA software package. A multi-layered structure was used to this end, with a first layer representing the surface contamination, followed by two layers representing the deposited film, a subsequent layer representing the interface between the substrate and the film, and a final layer accounting for the Si substrate.

The simulation data revealed the first layer to have 45–50 × 10^15^ atoms/cm^2^ (giving a thickness of ~4–6 nm) and an approximate Fe_0.12_C_0.15_H_0.15_O_0.58_ stoichiometry, indicating both a substantial contamination of oxidizing species (e.g., Fe_2_O_3_) as well as the presence of absorbed water molecules on the film surface. After exposure to air, the as-deposited Fe films quickly developed a nanometric layer of protective oxide [56] that can account for part of the surface contamination. Additionally, surface contamination can also occur during ion beam measurements [57].

Moreover, the simulation data of the NRBS spectra revealed a very thin layer corresponding to the interface between the substrate and the Fe film, of about 2–7 × 10^15^ atoms/cm^2^ SiO_2_ (~1–3.5 nm thick). This layer was characterized by a lower amount of oxygen compared to stoichiometric SiO_2_. At this point, it becomes important to highlight that the data analysis was performed by correlating the stoichiometry obtained from the Foil-ERDA and NRBS measurements. In other words, the final composition of each layer was constructed by using the input from each IBA technique, namely hydrogen concentration and the remaining elements from Foil-ERDA and NRBS, respectively. The results of the Foil-ERDA analysis showed a hydrogen content less than 1 at. % in all of the examined films.

The NRBS spectra for some representative Fe films are given on the left side of Figure 7 for the 3.043 MeV He beam, with different deposition parameters: RF source power (50 W, 100 W, and 150 W) in Figure 7a, deposition pressure (3 × 10^−3^ mbar Ar, 6 × 10^−3^ mbar Ar and 1 × 10^−2^ mbar Ar) in Figure 7b and target-to-substrate distance (13 cm and 10 cm) in Figure 7c. At this energy, the upper part of the films was probed. The elemental composition distribution over the lower half of the films is presented in Figure 7d–f, after probing the sample with the 3.075 MeV He beam. As indicated by the results presented in Table 3 and Figure 7, there is an influence of the deposition parameters on the film oxygen composition and its distribution over the film thickness. Furthermore, most of the films showed a non-uniform distribution of oxygen, with a higher concentration present in the upper part of the films. However, all of the Fe films grown at a deposition pressure P_d_ ≤ 3 × 10^−3^ mbar Ar and a RF source power P_s_ ≥ 125 W showed a uniform depth profile distribution of oxygen. This could indicate that, at least partially, the oxygen in the Fe films might arise from the interaction between the deposited Fe surface and the oxygen from the plasma, a process enhanced by increasing the deposition pressure and/or by decreasing the deposition rate. The NRBS data showed that by properly controlling the deposition conditions, Fe thin films with an oxygen concentration ≤1 at. % can be fabricated via RF sputtering.

## 4. Conclusions

The oblique angle RF sputtering growth of nanocolumnar Fe thin films on (100) Si templates was studied at room temperature for different RF source powers, deposition pressures and target-to-substrate distances. There are several conclusions that can be drawn from this study:

- According to XRD data the films grown following a (110) preferential orientation, the degree of polycrystallinity being dependent mainly on the average angle of incidence of the adatoms arriving on the surface; average crystallite size on (110) growth direction was calculated to be in the 15–25 nm range;
- HR-TEM, SEM and AFM studies showed that, due to the oblique growth configuration and the limited adatom surface diffusion, the films presented a nanocrystalline structure featuring tilted nanocolumns, with tilt angles in the 27–34° range (for the analyzed samples);
- The average islands size was in the 20–50 nm range, the films roughness increasing mainly with deposition pressure;
- The oxygen composition and distribution within the films, studied by means of EDS and NRBS, showed a non-uniform distribution with thickness in most of the films. However, uniform distribution of oxygen could be obtained when the growth took place under a narrow window of deposition conditions, i.e., a low deposition pressure (≤3 × 10^−3^ mbar Ar), combined with a high RF source power (≥125 W).
- The microstructural, morphological, and spectroscopic results showed that, once the deposition parameters have been optimized, there is a real possibility for RF sputtering to allow the fabrication of polycrystalline Fe films with desirable properties, such as a preferential orientation, a controllable surface topography and/or a very low concentration in impurities (such as oxygen), uniformly distributed throughout the film.

The Fe thin films resulting from such optimal depositions, having tunable nanocrystalline structures, could then be used as targets in order to enhance the laser-driven acceleration of ions for yielding ionizing beams. The manipulation of Fe target characteristics through the fabrication process may provide the flexibility necessary to obtain upon interaction with high-intensity ultrashort laser pulses an Fe beam with broad energy spectrum, that will better mimic the space radiation one.

## Figures and Tables

**Table 1:** Deposition parameters for the Fe thin films grown on (100) Si substrates, where P_s_—RF source power, P_d_—deposition pressure, and d_ts_—target-to-substrate distance.

Sample Parameter	S1	S2	S3	S4	S5	S6	S7	S8	S9
P_s_ (W)	50	100	150	125	125	125	125	125	125
P_d_ (mbar Ar)	6 × 10^−3^	6 × 10^−3^	6 × 10^−3^	1 × 10^−3^	3 × 10^−3^	6 × 10^−3^	1 × 10^−2^	3 × 10^−3^	3 × 10^−3^
d_ts_ (cm)	13	13	13	13	13	13	13	10	16

**Table 2:** Structural parameters of the Fe thin films grown on (100) Si substrates under different conditions. *a*—unit cell parameter for the (110) peak position, D_ef_—crystallite size in the (110) growth direction; A_(hkl)_—selected peak area; A_total_ = ΣA_(hkl)_, where (hkl) were (110), (200), and (211); eps—microscopic stress determined from (110) peak; ε_rel_—macroscopic stress determined from (110) peak. The fitting uncertainties for parameter *a* is ~0.006%, for D_ef_ and eps parameters is ~10%, while for ε_rel_ is ~3%.

Sample Name	*a* (Å)	D_ef_ (nm)	A_(200)_/A_total_	A_(211)_/A_total_	eps	|ε_rel_|
S1	2.854	15.2	0.108	0.178	3.43 × 10^−3^	1.8 × 10^−3^
S2	2.855	17.5	0.099	0.173	4.16 × 10^−3^	1.65 × 10^−3^
S3	2.856	20.6	0.093	0.166	4.20 × 10^−3^	1.25 × 10^−3^
S4	2.863	16.8	0.099	0.179	2.96 × 10^−3^	1.0 × 10^−3^
S5	2.861	18.2	0.097	0.176	3.63 × 10^−3^	5.0 × 10^−4^
S6	2.855	21.2	0.095	0.171	3.68 × 10^−3^	1.4 × 10^−3^
S7	2.855	24.7	0.055	0.139	3.96 × 10^−3^	1.6 × 10^−3^
S8	2.859	23.2	0.138	0.180	3.79 × 10^−3^	3.5 × 10^−4^

**Table 3:** EDS and NRBS analysis results for the deposited Fe films. Legend: at. % (EDS)—films elemental composition from EDS measurements; at. % (NRBS)—films elemental composition from NRBS measurements; td_NRBS_—films thickness distribution in TFU; t_NRBS_—films thickness from NRBS measurements. The measurements uncertainty: <1 at. %, for EDS, and <3 at. %, for NRBS.

Sample	Variable Parameter	at. % (EDS)	at. % (NRBS)	td_NRBS_ (10^15^ atoms/cm^2^)	t_NRBS_ (nm)
S1	Ps = 50 W	Fe_0.91_O_0.090_	(i) Fe_0.938_O_0.062_ (ii) Fe_0.965_O_0.035_	(i) 425 (ii) 425	110
S2	P_s_ = 100 W	Fe_0.928_O_0.072_	(i) Fe_0.94_O_0.06_ (ii) Fe_0.985_O_0.015_	(i) 300 (ii) 710	129
S3	P_s_ = 150 W	Fe_0.94_O_0.060_	(i) Fe_0.955_O_0.045_ (ii) Fe_0.98_O_0.02_	(ii) 450 (iii) 450	116
S4	P_d_ = 1 × 10^−3^ mbar	Fe_0.962_O_0.038_	(i) Fe_0.99_O_0.01_ (ii) Fe_0.99_O_0.01_	(i) 450 (ii) 450	116
S5	P_d_ = 3 × 10^−3^ mbar	Fe_0.942_O_0.058_	(i) Fe_0.99_O_0.01_ (ii) Fe_0.99_O_0.01_	(i) 405 (ii) 440	109
S6	P_d_ = 6 × 10^−3^ mbar	Fe_0.923_O_0.077_	(i) Fe_0.95_O_0.05_ (ii) Fe_0.98_O_0.02_	(i) 355 (ii) 500	110
S7	P_d_ = 1 × 10^−2^ mbar	Fe_0.89_O_0.11_	(i) Fe_0.90_O_0.10_ (ii) Fe_0.95_O_0.05_	(i) 395 (ii) 395	102
S8	d_ts_ = 10 cm	Fe_0.939_O_0.061_	(i) Fe_0.978_O_0.022_ (ii) Fe_0.978_O_0.022_	(i) 315 (ii) 322	85

## Data Availability

Not applicable.

## References

[1] Gales S., Balabanski D.L., Negoita F., Tesileanu O., Ur C., Ursescu D., Zamfir N. (2016). New frontiers in nuclear physics with high-power lasers and brilliant monochromatic gamma beams. Phys. Scr..

[2] Durante M., Golubev A., Park W.-Y., Trautmann C. (2019). Applied nuclear physics at the new high-energy particle accelerator facilities. Phys. Rep..

[3] Yogo A., Sato K., Nishikino M., Mori M., Teshima T., Numasaki H., Murakami M., Demizu Y., Akagi S., Nagayama S. (2009). Application of laser-accelerated protons to the demonstration of DNA double-strand breaks in human cancer cells. Appl. Phys. Lett..

[4] Kraft S.D., Richter C., Zeil K., Baumann M., Beyreuther E., Bock S., Bussmann M., Cowan T.E., Dammene Y., Enghardt W. (2010). Dose-dependent biological damage of tumour cells by laser-accelerated proton beams. New J. Phys..

[5] Doria D., Kakolee K.F., Kar S., Litt S.K., Fiorini F., Ahmed H., Green S., Jeynes J.C.G., Kavanagh J., Kirby D. (2012). Biological effectiveness on live cells of laser driven protons at dose rates exceeding 109 Gy/s. AIP Adv..

[6] Ledingham K., Galster W. (2010). Laser-driven particle and photon beams and some applications. New J. Phys..

[7] Borghesi M., Bigongiari A., Kar S., Macchi A., Romagnani L., Audebert P., Fuchs J., Toncian T., Willi O., Bulanov S. (2008). Laser-driven proton acceleration: Source optimization and radiographic applications. Plasma Phys. Control. Fusion.

[8] Brenner C., Mirfayzi S., Rusby D., Armstrong C., Alejo A., Wilson L., Clarke R., Ahmed H., Butler N., Haddock D. (2016). Laser-driven x-ray and neutron source development for industrial applications of plasma accelerators. Plasma Phys. Control. Fusion.

[9] Daido H., Nishiuchi M., Pirozhkov A.S. (2012). Review of laser-driven ion sources and their applications. Reports Prog. Phys..

[10] Asavei T., Tomut M., Bobeica M., Aogaki S., Cernaianu M., Ganciu M., Kar S., Manda G., Mocanu N., Neagu L. (2016). Materials in extreme environments for energy, accelerators and space applications at ELI-NP. Rom. Reports Phys..

[11] Bobeica M., Aogaki S., Asavei T., Cernaianu M.O., Ghenuche P., Stutman D. (2018). Dose calculations in a cell monolayer for high-throughput irradiation with proton beams generated by PW lasers for space applications. Life Sci. Sp. Res..

[12] Onorato G., Di Schiavi E., Di Cunto F. (2020). Understanding the Effects of Deep Space Radiation on Nervous System: The Role of Genetically Tractable Experimental Models. Front. Phys..

[13] Norbury J.W., Schimmerling W., Slaba T.C., Azzam E.I., Badavi F.F., Baiocco G., Benton E., Bindi V., Blakely E.A., Blattnig S.R. (2016). Galactic cosmic ray simulation at the NASA Space Radiation Laboratory. Life Sci. Sp. Res..

[14] Chew M.T., Nisbet A., Jones B., Suzuki M., Matsufuji N., Murakami T., Bradley D.A. (2019). Ion beams for space radiation radiobiological effect studies. Radiat. Phys. Chem..

[15] Gales S., Tanaka K.A., Balabanski D.L., Negoita F., Stutman D., Tesileanu O., Ur C.A., Ursescu D., Andrei I., Ataman S. (2018). The extreme light infrastructure—nuclear physics (ELI-NP) facility: New horizons in physics with 10 PW ultra-intense lasers and 20 MeV brilliant gamma beams. Reports Prog. Phys..

[16] Cucinotta F.A., Alp M., Sulzman F.M., Wang M. (2014). Space radiation risks to the central nervous system. Life Sci. Sp. Res..

[17] Asaithamby A., Chen D.J. (2011). Mechanism of cluster DNA damage repair in response to high-atomic number and energy particles radiation. Mutat. Res. Mol. Mech. Mutagen..

[18] McKenna P., Ledingham K.W.D., Yang J.M., Robson L., McCanny T., Shimizu S., Clarke R.J., Neely D., Spohr K., Chapman R. (2004). Characterization of proton and heavier ion acceleration in ultrahigh-intensity laser interactions with heated target foils. Phys. Rev. E.

[19] Nishiuchi M., Sakaki H., Esirkepov T.Z., Nishio K., Pikuz T.A., Faenov A.Y., Skobelev I.Y., Orlandi R., Sako H., Pirozhkov A.S. (2015). Acceleration of highly charged GeV Fe ions from a low-Z substrate by intense femtosecond laser. Phys. Plasmas.

[20] Hegelich B.M., Albright B.J., Cobble J., Flippo K., Letzring S., Paffett M., Ruhl H., Schreiber J., Schulze R.K., Fernández J.C. (2006). Laser acceleration of quasi-monoenergetic MeV ion beams. Nature.

[21] Prencipe I., Fuchs J., Pascarelli S., Schumacher D.W., Stephens R.B., Alexander N.B., Briggs R., Büscher M., Cernaianu M.O., Choukourov A. (2017). Targets for high repetition rate laser facilities: Needs, challenges and perspectives. High Power Laser Sci. Eng..

[22] Ceccotti T., Floquet V., Sgattoni A., Bigongiari A., Raynaud M., Riconda C., Heron A., Baffigi F., Labate L., Gizzi L. (2013). Evidence of resonant surface wave excitation in the relativistic regime through measurements of proton acceleration from grating targets. Phys. Rev. Lett..

[23] Gheorghiu C.C., Cerchez M., Aktan E., Prasad R., Yilmaz F., Yilmaz N., Popa D., Willi O., Leca V. (2022). Fabrication of micrometre-sized periodic gratings in free-standing metallic foils for laser–plasma experiments. High Power Laser Sci. Eng..

[24] Habara H., Honda S., Katayama M., Sakagami H., Nagai K., Tanaka K.A. (2016). Efficient energy absorption of intense ps-laser pulse into nanowire target. Phys. Plasmas.

[25] Zhao Z., Cao L., Cao L., Wang J., Huang W., Jiang W., He Y., Wu Y., Zhu B., Dong K. (2010). Acceleration and guiding of fast electrons by a nanobrush target. Phys. Plasmas.

[26] Prencipe I., Sgattoni A., Dellasega D., Fedeli L., Cialfi L., Choi I.W., Kim I.J., Janulewicz K.A., Kakolee K.F., Lee H.W. (2016). Development of foam-based layered targets for laser-driven ion beam production. Plasma Phys. Control. Fusion.

[27] Margarone D., Klimo O., Kim I.J., Prokůpek J., Limpouch J., Jeong T.M., Mocek T., Pšikal J., Kim H.T., Proška J. (2012). Laser-Driven Proton Acceleration Enhancement by Nanostructured Foils. Phys. Rev. Lett..

[28] Gheorghiu C.C., Ionescu S.C., Ghenuche P., Cernaianu M.O., Doria D., Popa D., Leca V. (2021). Structuring Free-Standing Foils for Laser-Driven Particle Acceleration Experiments. Front. Phys..

[29] Dalui M., Wang W.-M., Trivikram T.M., Sarkar S., Tata S., Jha J., Ayyub P., Sheng Z.M., Krishnamurthy M. (2015). Preferential enhancement of laser-driven carbon ion acceleration from optimized nanostructured surfaces. Sci. Rep..

[30] Barranco A., Borras A., Gonzalez-Elipe A.R., Palmero A. (2016). Perspectives on oblique angle deposition of thin films: From fundamentals to devices. Prog. Mater. Sci..

[31] Hawkeye M.M., Brett M.J. (2007). Glancing angle deposition: Fabrication, properties, and applications of micro- and nanostructured thin films. J. Vac. Sci. Technol. A.

[32] Bairagi S., Järrendahl K., Eriksson F., Hultman L., Birch J., Hsiao C.-L. (2020). Glancing Angle Deposition and Growth Mechanism of Inclined AlN Nanostructures Using Reactive Magnetron Sputtering. Coatings.

[33] Zhou C., Li T., Wei X., Yan B. (2020). Effect of the Sputtering Power on the Structure, Morphology and Magnetic Properties of Fe Films. Metals.

[34] Gheorghiu C.C., Leca V., Popa D., Cernaianu M.O., Stutman D. (2016). Overview on the target fabrication facilities at ELI-NP and ongoing strategies. J. Instrum..

[35] Gallego J.M., Miranda R. (1991). The Fe/Si(100) interface. J. Appl. Phys..

[36] Horcas I., Fernández R., Gómez-Rodríguez J.M., Colchero J., Gómez-Herrero J., Baro A.M. (2007). WSXM: A software for scanning probe microscopy and a tool for nanotechnology. Rev. Sci. Instrum..

[37] Ene V.L., Dinescu D., Zai I., Djourelov N., Vasile B.S., Serban A.B., Leca V., Andronescu E. (2019). Study of edge and screw dislocation density in GaN/Al2O3 heterostructure. Materials.

[38] Burducea I., Straticiuc M., Ghiță D.G., Moșu D.V., Călinescu C.I., Podaru N.C., Mous D.J.W., Ursu I., Zamfir N.V. (2015). A new ion beam facility based on a 3MV Tandetron^TM^ at IFIN-HH, Romania. Nucl. Instrum. Methods Phys. Res. Sect. B Beam Interact. Mater. At..

[39] Mayer M. (1999). SimNRA, a simulation program for the analysis of NRA, RBS and ERDA. AIP Conf. Proc..

[40] Ion L., Iftimie S., Radu A., Antohe V.A., Toma O., Antohe S. (2021). Physical properties of RF-sputtered ZnSe thin films for photovoltaic applications: Influence of film thickness. Proc. Rom. Acad. Ser. A.

[41] Toma O., Ion L., Iftimie S., Antohe V.A., Radu A., Raduta A.M., Manica D., Antohe S. (2019). Physical properties of rf-sputtered ZnS and ZnSe thin films used for double-heterojunction ZnS/ZnSe/CdTe photovoltaic structures. Appl. Surf. Sci..

[42] Rodriguez-Carvajal J. (2003). Study of micro-structural effects by powder diffraction using the program FULLPROF. Lab. Léon Brillouin (CEA-CNRS) CEA/Saclay.

[43] Gheisari K., Ong C.K. (2020). Magnetic properties and thermal stability of nanocrystalline Fe films prepared by oblique sputtering deposition method. Phys. B Condens. Matter.

[44] Javed A., Morley N.A., Gibbs M.R.J. (2011). Effect of growth parameters on the structure and magnetic properties of thin polycrystalline Fe films fabricated on Si〈100〉 substrates. Appl. Surf. Sci..

[45] Hull A.W. (1917). A New Method of X-Ray Crystal Analysis. Phys. Rev..

[46] Okamoto K., Itoh K. (2005). Incidence angle dependences of columnar grain structure and texture in obliquely deposited iron films. Jpn. J. Appl. Phys..

[47] Okamoto K., Hashimoto T., Itoh K., Hara K., Kamiya M., Fujiwara H. (1995). Crystallographic contribution to the formation of columnar grain structure in iron films deposited at oblique incidence. J. Magn. Magn. Mater..

[48] Nieuwenhuizen J.M., Haanstra H.B. (1966). Microfractography of thin films. Philips Tech. Rev..

[49] Lichter S., Chen J. (1986). Model for Columnar Microstructure of Thin Solid Films. Phys. Rev. Lett..

[50] Meakin P. (1988). Ballistic deposition onto inclined surfaces. Phys. Rev. A.

[51] Tait R.N., Smy T., Brett M.J. (1993). Modelling and characterization of columnar growth in evaporated films. Thin Solid Films.

[52] Gavrilova T.A., Atuchin V.V., Kruchinin V.N., Lychagin D.V. (2012). Micromorphology and spectroscopic ellipsometry of Ni(100) crystal surface. Phys. Procedia.

[53] Atuchin V.V., Kochubey V.A., Kozhukhov A.S., Kruchinin V.N., Pokrovsky L.D., Soldatenkov I.S., Troitskaia I.B. (2019). Microstructure and dispersive optical parameters of iron films deposited by the thermal evaporation method. Optik.

[54] Nastasi N., Mayer J., Wang Y. (2015). Ion Beam Analysis: Fundamentals and Applications.

[55] Jeynes C. (2012). Elastic Backscattering of Ions for Compositional Analysis. Charact. Mater..

[56] Baran J.D., Grönbeck H., Hellman A. (2014). Mechanism for Limiting Thickness of Thin Oxide Films on Aluminum. Phys. Rev. Lett..

[57] Velişa G., Trocellier P., Thomé L., Vaubaillon S., Sattonnay G., Miro S., Serruys Y. (2014). Patterning SiC nanoprecipitate in Si single crystals by simultaneous dual- beam ion implantation. J. Mater. Sci..

